# Case Report: Effective management of residual glioblastoma with combined modality therapy

**DOI:** 10.3389/fonc.2025.1451408

**Published:** 2025-03-28

**Authors:** Heng-Jui Chang, Chiao-Hsu Ke, Yu-Shan Wang, Yu-Cheng Kuo

**Affiliations:** ^1^ Department of Radiation Oncology, Wesing Surgery Hospital, Kaohsiung, Taiwan; ^2^ Department of Chemical Engineering and Biotechnology, Institute of Chemical Engineering, National Taipei University of Technology, Taipei, Taiwan; ^3^ Department of Veterinary Medicine, School of Veterinary Medicine, National Taiwan University, Taipei, Taiwan; ^4^ Department of Research and Development, Uni-Pharma Co-Ltd., Taipei, Taiwan; ^5^ Department of Radiation Oncology, China Medical University Hsinchu Hospital, Hsinchu, Taiwan; ^6^ School of Medicine, China Medical University, Taichung, Taiwan

**Keywords:** glioblastoma, oncothermia, modulated electro-hyperthermia, cancer management, brain tumor

## Abstract

Glioblastoma (GBM) is an aggressive primary brain tumor with a poor prognosis, often characterized by rapid progression and resistance to conventional therapies. This case report discusses the comprehensive management of a 60-year-old female diagnosed with residual GBM following initial surgical intervention. The treatment regimen included craniectomy, concurrent chemoradiotherapy (CCRT), adjuvant temozolomide, and weekly sessions of modulated electro-hyperthermia (mEHT, Oncothermia). Remarkably, the patient exhibited significant tumor shrinkage, improved neurological symptoms, and an extended survival period compared to typical outcomes. mEHT was utilized to enhance the efficacy of chemoradiotherapy and temozolomide by selectively targeting cancer cells and improving drug delivery. Integrating mEHT into the standard treatment protocol appears to have contributed to better therapeutic outcomes and improved quality of life for the patient. This case underscores the potential benefits of incorporating mEHT into multimodal treatment strategies for GBM, highlighting its role in enhancing the effects of conventional therapies. Future research and clinical trials are warranted to further explore the synergistic effects of mEHT with standard GBM treatments, aiming to establish more effective protocols and improve overall patient survival and quality of life. This report adds to the growing body of evidence supporting the use of innovative, integrative approaches in the management of aggressive brain tumors like GBM.

## Introduction

Glioblastoma (GBM) is the most common and aggressive primary malignant brain tumor in adults, accounting for approximately 47.7% of all primary malignant brain tumors (1). Despite advances in surgical techniques, radiotherapy, and chemotherapy, the prognosis for patients with GBM remains poor, with a median overall survival of 15 months following diagnosis (2). Standard treatment involves maximal safe resection followed by concurrent chemoradiotherapy (CCRT) with temozolomide (TMZ) and adjuvant temozolomide therapy. However, GBM’s highly invasive nature, resistance to conventional treatments, and frequent recurrence pose significant challenges in achieving long-term control (3).

Modulated electro-hyperthermia (mEHT, Oncothermia) is an emerging treatment modality that uses low-intensity radiofrequency to selectively target cancer cells, enhancing the efficacy of chemotherapy and radiotherapy. By inducing cell death through apoptosis and mitotic catastrophe, mEHT enhances the damage caused by DNA-damaging agents (4, 5). mEHT operates by heating tumor tissues to approximately 40–42°C, which disrupts the cancer cells’ ability to repair DNA damage, making them more vulnerable to concurrent therapies (6). Its use as an adjunct therapy in GBM has shown promising results in preclinical and clinical studies, with evidence suggesting that it may improve tumor control and prolong survival (7). mEHT has emerged as a promising adjunctive therapy in the treatment of various malignancies, including glioblastoma (GBM). Unlike conventional hyperthermia, which relies on the homogeneous heating of tumors, mEHT selectively targets malignant cells by exploiting the differences in electrical properties between cancerous and normal tissues. This selective heating leads to increased apoptosis, immune modulation, and improved tumor perfusion, enhancing the efficacy of chemotherapy and radiotherapy. Clinical evidence supports the efficacy of mEHT in managing recurrent GBM, with studies demonstrating improved tumor control and survival outcomes.

The safety and efficacy of mEHT depend on optimized power delivery, ensuring that cancer cells are effectively targeted while sparing healthy brain tissue. Clinical studies have demonstrated that power levels up to 80 watts are safe for brain tumors, maintaining normal brain temperatures below 39.2°C while effectively increasing tumor perfusion and sensitizing cancer cells to radiation and chemotherapy (8, 9).

A retrospective multicenter controlled study by Fiorentini et al. (10) found that mEHT, when used in combination with integrative cancer therapies, contributed to prolonged survival and improved quality of life in patients with relapsed malignant glioblastoma and astrocytoma. While most evidence pertains to recurrent GBM, mEHT has shown potential as an adjunct therapy in newly diagnosed GBM, as it increases the sensitivity of cancer cells to standard treatments, potentially leading to better outcomes. Further investigation is warranted to confirm its efficacy in newly diagnosed cases​ (7). These findings support the potential of mEHT as an adjunct treatment in recurrent GBM, but there is limited data on its use in newly diagnosed cases.

In this report, we present a unique case of newly diagnosed GBM treated with a combination of standard CCRT and mEHT. This case highlights the potential for mEHT to enhance treatment outcomes in the initial management of GBM, rather than only in the recurrent setting. To our knowledge, this is the first case report documenting the use of mEHT in a newly diagnosed GBM patient, and it opens the door for further investigation into the efficacy of this treatment approach in earlier stages of disease.

## Case presentation

A 60-year-old female was diagnosed with GBM and underwent craniectomy and tumor removal in March 2023. However, after surgery, some tumor tissue remained. The patient received comprehensive treatment, including radiotherapy, chemotherapy, and mEHT. She has a medical history of dyslipidemia but no history of diabetes mellitus (DM), hypertension (HTN), hepatitis B virus (HBV), or hepatitis C virus (HCV). The patient occasionally consumes alcohol but does not smoke or chew betel nut. Additionally, there is no family history of cancer.

The patient is right-hand dominant. The symptoms began on January 22, 2023, with initial left palm numbness, lack of sensation in the fingers, a shooting-like sensation on the left side of the face, and neck pain. These symptoms lasted 5-10 minutes and recurred every 2-3 days.

Neurologically, the patient’s right-hand muscle power was assessed as 5/5, and the left-hand muscle power was 4/5. Cranial nerves were intact, and the patient’s Judgment, Orientation, Memory, Attention, and Calculation (JOMAC) were stable. There was no evidence of trunk ataxia, and all deep tendon reflexes (DTR) were normal.

**Figure 1:**
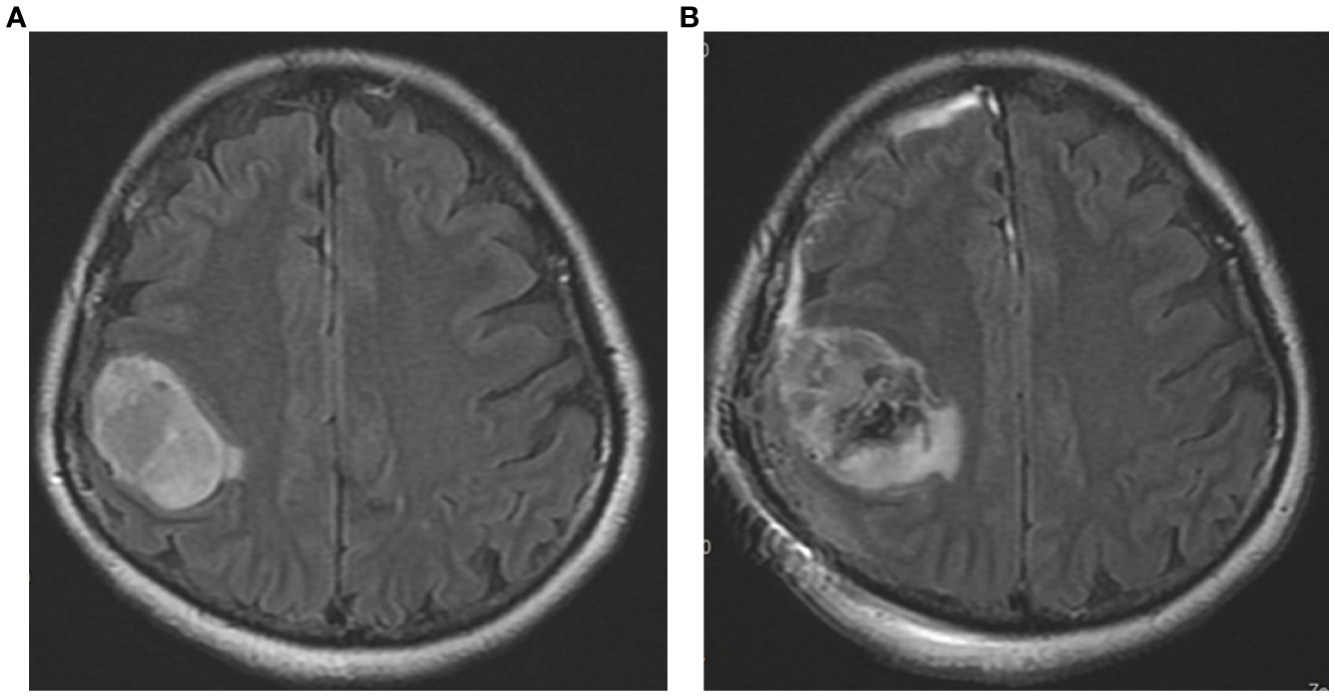
The pre-operative and post-operative MRI images of the patient’s brain taken in T2/FLAIR sequences. **(A)** The preoperative image (dated 2023/3/7) shows a large tumor mass in the right parietal region. **(B)** The postoperative image (dated 2023/3/14) depicts the condition after surgical resection.

The patient was diagnosed with glioblastoma after an MRI on March 7, 2023, and had surgery on March 13, 2023. Although the surgery reduced some of the tumor volume, a substantial portion of the tumor remains visible in the resection cavity, suggesting an incomplete resection. The surrounding brain tissue exhibited significant edema, which was anticipated after the surgery and addressed in the subsequent treatment plan. **Figure 1** illustrated that while the tumor resection reduced part of the tumor burden, a large residual tumor was still present, which was addressed through follow-up treatments, including chemoradiotherapy and hyperthermia. This highlights the necessity of continued multimodal treatment to control the remaining disease.

Before the surgery, the patient’s Karnofsky Performance Scale (KPS) was around 80. Following the surgery, up until the MRI scan in June 2023, the patient experienced persistent swelling, and her KPS was around 50-60. However, from June to October 2023, the MRI showed a reduction in the swelling and continued shrinkage of the tumor, leading to an improvement in the KPS back to 80. By January 2024, MRI results indicated that the swelling had almost entirely resolved, and the patient’s KPS had improved to 90. The primary remaining symptom at this time was numbness in the left palm, but the muscle power in the affected hand had returned to a score of 5.

The patient had surgery on March 13, 2023, and was discharged on May 12, 2023. During her time in the hospital, the patient received radiotherapy and oral chemotherapy at the same time. She did not take steroids and did not have any seizure attacks. However, she was given preventive medications during and after her hospital stay, which included duloxetine 30 mg twice daily (BID), haloperidol 0.5 mg at bedtime (HS), quetiapine 25 mg at bedtime (HS), and levetiracetam 500 mg twice daily (BID).

Histologic Type and Location: The patient had a glioblastoma, IDH-wildtype (WHO Grade IV), located in the right parietal lobe.

Immunohistochemical Stains (IHC): The stains showed GFAP (+), IDH-1 (-), intact ATRX, and no significant overexpression of p53. However, data on MGMT methylation and NGS are not available.

Microscopic Description: The section showed widespread glial cell proliferation with increased cell density, abnormal cells, and significant mitosis. Necrosis and glomeruloid vascular proliferation were also observed.

Prognostic and Predictive Factors: Histologic grade: WHO Grade IV. The surgical margins could not be assessed, so they are considered positive.

## Radiotherapy

**Figure 2:**
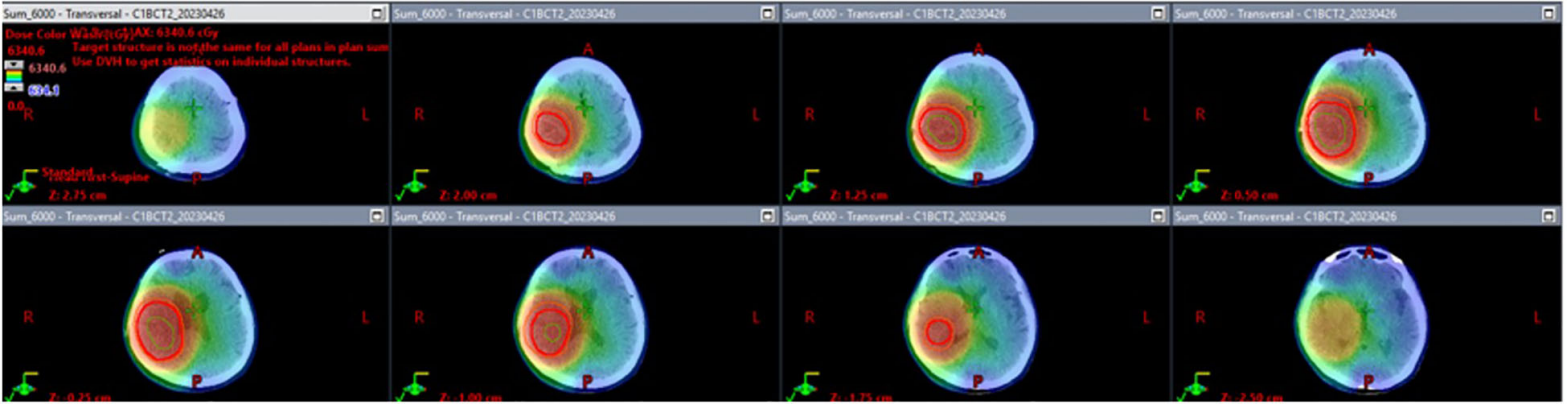
Radiation field mapping of glioblastoma treatment plan. The image displays radiation field mapping for the patient’s radiotherapy treatment, showing how the radiation dose is distributed across the brain to target the tumor. The key details include the Gross Tumor Volume (GTV), Clinical Target Volume (CTV), and Planning Target Volume (PTV). The total prescribed dose is 6000 cGy, with color gradients indicating how the dose tapers off around the tumor, ensuring effective targeting of tumor cells while minimizing exposure to healthy tissues.

In **Figure 2**, the radiation field mapping for the patient’s treatment plan is illustrated. Each panel represents a cross-sectional image from a CT scan, showing the distribution of radiation dose across the targeted area. **Figure 1** displays the planned dose for the patient’s radiotherapy, with different colors representing different dose levels. Red marks the region receiving the full prescribed dose, while the surrounding color gradients indicate the gradual fall-off of the dose to nearby tissues. In **Figure 1**, the dose was modulated to minimize radiation exposure to critical structures surrounding the tumor while ensuring that the tumor receives the intended therapeutic dose. The total dose delivered was 6000 cGy across the region of interest, including the tumor and margins necessary to ensure adequate tumor control.

The patient began radiotherapy on March 30, 2023, and completed it on May 11, 2023. She received a total of 30 fractions with a cumulative dose of 6000 cGy. The radiotherapy was carried out in two phases. In the first phase, a 2-centimeter margin was added to the clinical target volume (CTV) to accommodate peripheral edema, and a dose of 4600 cGy was administered. In the second phase, the treatment area was reduced to specifically target the residual tumor area without edema, and an additional boost of 1400 cGy was given, resulting in a total dose of 6000 cGy. No steroid was used during radiotherapy.

**Figure 3:**
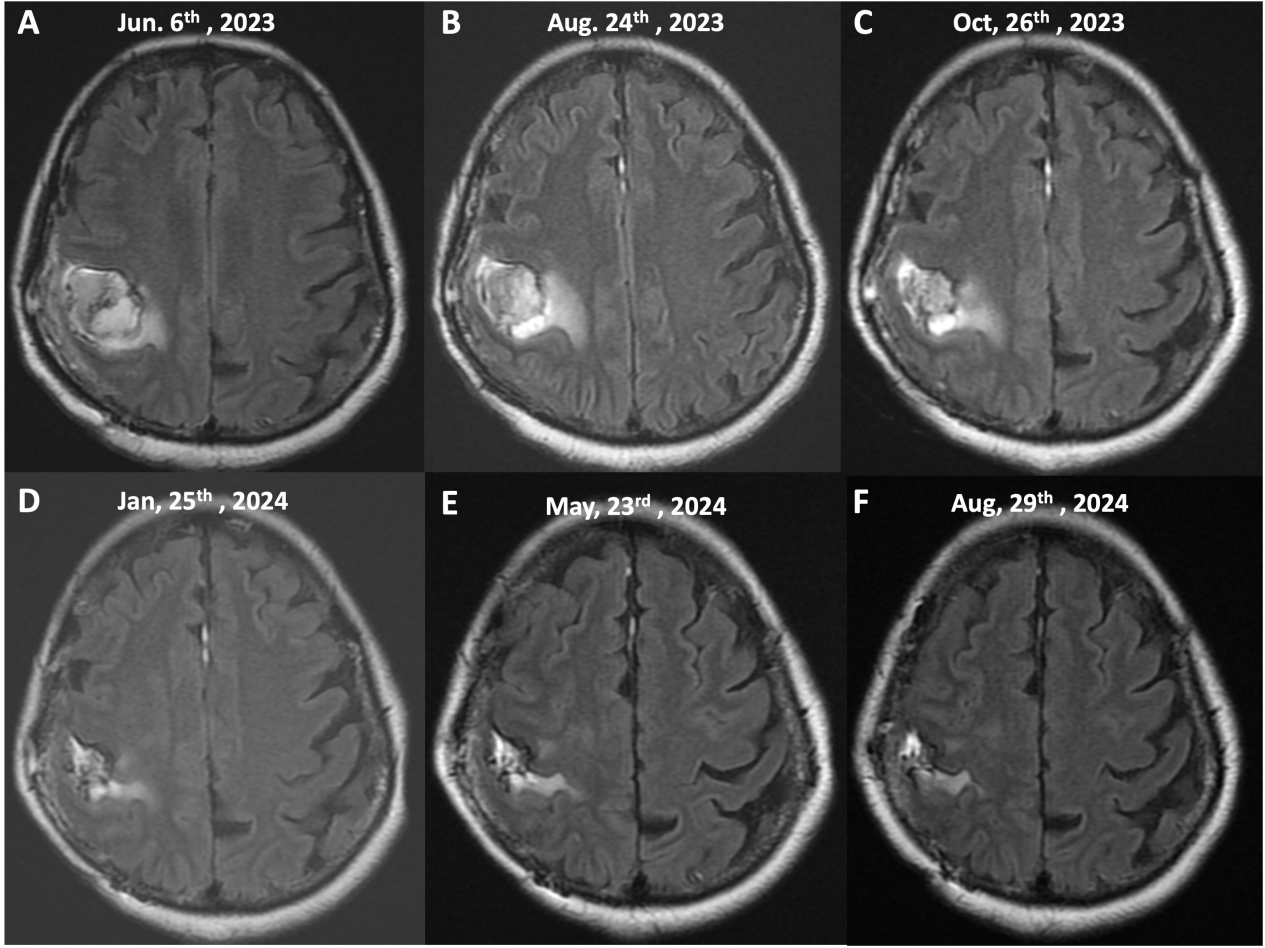
Serial MRI imaging of tumor progression and response to treatment (T2/FLAIR Sequences, Axial View). This figure illustrates the sequential MRI images of the patient’s brain taken over a 15-month period, demonstrating the tumor’s response to treatment, including chemoradiotherapy (CCRT) and continuous mEHT therapy. The images show axial T2/FLAIR sequences at different stages of the patient’s treatment course: **(A)** (June 6th, 2023), **(B)** (August 24th, 2023), **(C)** (October 26th, 2023), **(D)** (January 25th, 2024), **(E)** (May 23rd, 2024), **(F)** (August 29th, 2024).

**Figures 4:**
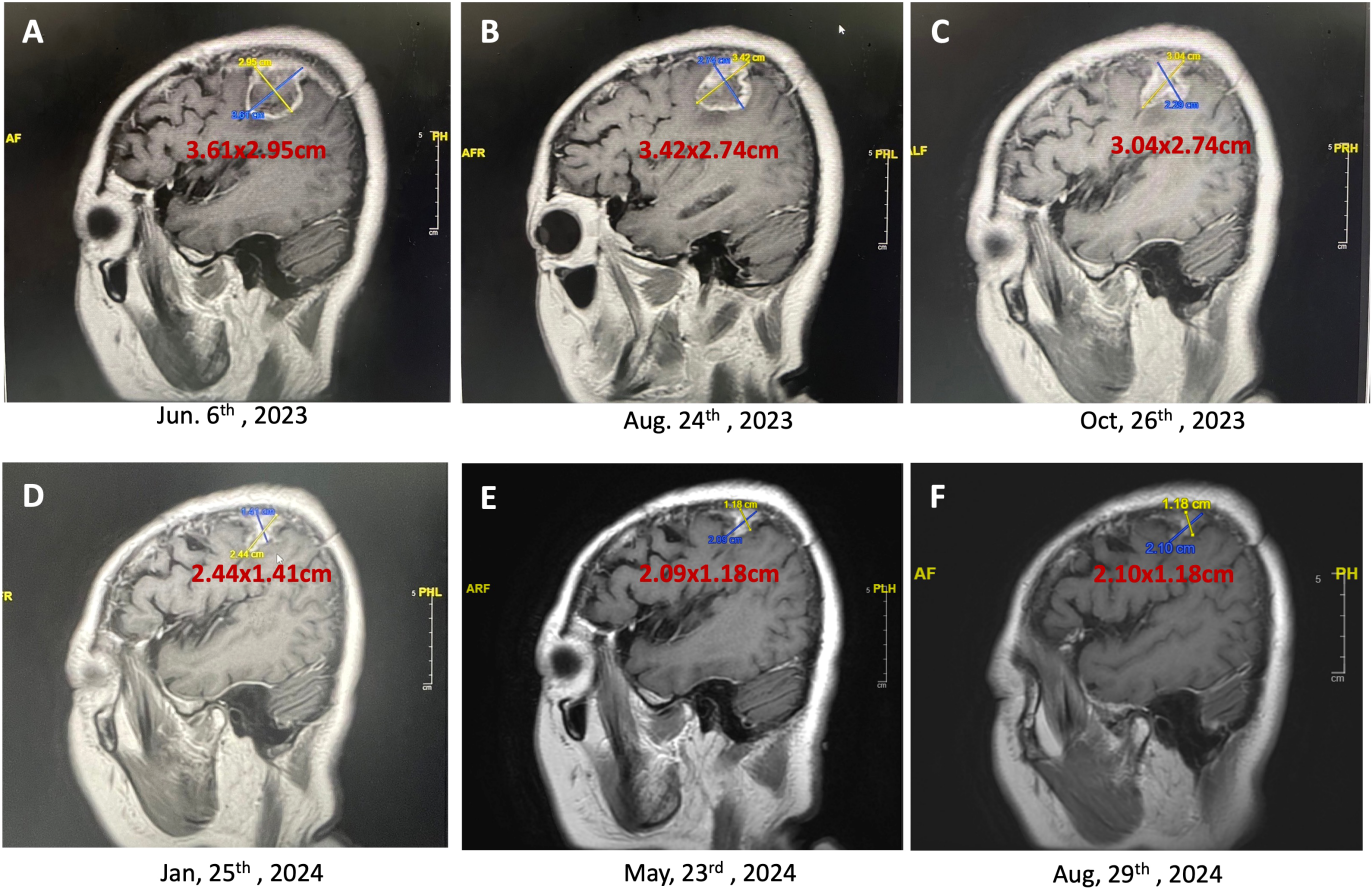
Persistent reduction in brain tumor size over time (T1 contrast-enhanced MRI, sagittal view). This series of MRI images demonstrated the continuing reduction in the size of a brain tumor in a 60-year-old female patient undergoing a combination of craniectomy, chemoradiotherapy (CCRT), adjuvant temozolomide, and weekly mEHT sessions. **(A)**, The initial MRI scan shows a tumor size of 3.61 x 2.95 cm (June 6, 2023); **(B)**, Following two months of treatment, the tumor size reduced to 3.42 x 2.74 cm (August 24, 2023); **(C)**, Further reduction in tumor size to 3.04 x 2.74 cm is observed (October 26, 2023); **(D)**, Continued treatment resulted in a significant reduction in tumor size to 2.44 x 1.41 cm (January 25, 2024); **(E)**, The latest MRI scan shows the tumor has shrunk to 2.09 x 1.18 cm (May 23, 2024), demonstrating the effectiveness of the multimodal treatment approach in reducing tumor size and improving patient prognosis. **(F)**, The final MRI shows the tumor size stabilizing at 2.10 x 1.18 cm (August 29, 2024) with minimal contrast enhancement, indicating the effectiveness of the ongoing mEHT therapy in maintaining disease stability.

The post-radiotherapy MRI image, taken on June 6th, 2023 (**Figures 3A**, **4A**), shows the tumor following concurrent chemoradiotherapy (CCRT) treatment. At this stage, the T2/FLAIR sequence reveals a decrease in tumor size with surrounding edema still present. The therapeutic response to radiotherapy is evident, as there is a reduction in the tumor’s intensity compared to the pre-treatment scans. However, the presence of residual tumor and edema suggests the need for continued monitoring and additional treatments.

## Chemotherapy

After undergoing radiotherapy, the patient was given a daily dose of 120 mg of Temozolomide. The patient’s body surface area (BSA) was calculated based on her height (161 cm) and weight (64 kg), resulting in a BSA of 1.62 m². According to standard dosing guidelines, she should have received a dose of 75 mg/m² of Temozolomide, which equals 121.5 mg daily. However, since Temozolomide is only available in 100 mg and 20 mg formulations, she was administered 120 mg daily during radiotherapy as part of the concurrent chemoradiotherapy (CCRT) protocol.

Following the completion of radiotherapy, the patient started an adjuvant Temozolomide regimen, taking 340 mg daily for 5 days every 28 days. A 200 mg/m² dose was given, equal to 330 mg daily. However, due to the available dosage forms (100 mg and 20 mg tablets), the daily dose was rounded to 340 mg. This post-radiation chemotherapy treatment began on July 6, 2023.

All the dates the patient received adjuvant Temozolomide were listed: 2023/6/7-6/11, 7/6-7/10, 8/1-8/5, 8/31-9/4, 9/28-10/2, 10/26-10/30, 11/23-11/27, 12/21-12/25, 2024/1/18-1/22, 2/15-2/19, 3/14-3/18, 4/11-4/15, 5/9-5/13, 6/6-6/10, 7/4-7/8, 8/1-8/5, and 8/29-9/2.

The patient continues this regimen as her disease remains stable without progression.

## mEHT

The patient underwent mEHT (Oncothermia) as part of her treatment once a week consistently without any breaks. Weekly mEHT sessions began on May 24, 2023, and as of September 12, 2024, 67 mEHT sessions had been completed. Each session lasted for 60 minutes. The mEHT machine used for the treatments was the EHY-2000 model, operating at a frequency of 13.56 MHz. The power output was kept mainly at 80 watts to target cancer cells while preserving healthy tissues.

The mEHT treatments were specifically administered to the site of the brain. This approach utilizes controlled electromagnetic fields to selectively increase the temperature of cancer cells, causing stress on their cell membranes and leading to cell death. This selective heating enhances the effectiveness of concurrent treatments, such as immunotherapy and chemotherapy, by making the cancer cells more susceptible to these therapies. The treatment was well-tolerated, and no significant adverse events or complications were reported during therapy. The peripheral edema and tumor both gradually reduced in size, so no steroids or Avastin (bevacizumab) were administered. **Figures 3**, **4** showed the effects of mEHT on the patient’s tumor size over time.

## Tumor status

Initial imaging post-CCRT and mEHT demonstrated a residual tumor measuring 3.61 x 2.95 cm. Subsequent imaging at thirteen months post-surgery revealed a significant reduction in tumor size to 2.10 x 1.18 cm. The tumor continued to shrink without associated edema.

## Symptoms and quality of life

The patient first experienced symptoms on January 22, 2023, which included numbness in the left palm, a lack of sensation in the fingers, a shooting-like sensation on the left side of the face (hemiface), and neck pain on the left side. These symptoms lasted 5-10 minutes before disappearing but would return every 2-3 days. Upon examination, hand muscle strength was rated at five on the right and four on the left. The patient’s cranial nerves were intact, their judgment and memory (JOMAC) were stable, no signs of trunk ataxia were present, and deep tendon reflexes (DTR) were normal.

An MRI scan confirmed the diagnosis of the tumor on March 7, 2023, and surgery was performed on March 13, 2023. Before surgery, the patient’s Karnofsky Performance Scale (KPS) score was approximately 80. Following the surgery, the MRI scans up until June 2023 still showed some edema; during this time, the KPS score dropped to 50-60. From June to October 2023, the MRI scans indicated a reduction in the edematous area, and the tumor continued to shrink, leading to an improvement in the patient’s KPS score to 80. By January 2024, the MRI showed that the edema had nearly completely resolved, and the patient’s KPS score returned to 90. The primary residual symptom was numbness in the left palm, but muscle strength had fully recovered to a score of 5.

A timeline of the patient’s symptoms and changes:

◆ 2023/1/22:
 ◆ Initial onset of symptoms, including numbness in the left palm, lack of sensation in the fingers, shooting pain on the left hemiface, and left neck pain.
 ◆ Symptoms persisted for 5-10 minutes and returned every 2-3 days.
 ◆ Hand muscle strength: right hand = 5/5, left hand = 4/5.
 ◆ Cranial nerves were intact, JOMAC stable, no trunk ataxia, DTRs were normal.
◆ 2023/3/7:
 ◆ MRI confirmed the diagnosis of the tumor.
◆ 2023/3/13:
◆ Surgery performed.
 ◆ Pre-surgery Karnofsky Performance Scale (KPS) score was approximately 80.
◆ 2023/3 – 2023/6:
 ◆ Post-surgery, MRI scans showed persistent edema.
◆ KPS score dropped to approximately 50-60 during this period.
◆ 2023/6 – 2023/10:
 ◆ MRI scans showed a reduction in the edematous area and continued tumor shrinkage.
 ◆ KPS improved to 80 during this time.
◆ 2024/1:
◆ MRI showed that the edema had almost completely resolved.
◆ KPS score returned to 90.
◆ Residual symptoms included left palm numbness, but hand muscle strength had fully recovered to a score of 5/5.

## Survival

The patient exhibited remarkable survival and disease control, with a stable disease state lasting 16 months following initial resection. While a 14-month survival post-resection may not be extraordinary by current standards, the key observation in this case is the extended progression-free interval (PFI) of 16 months. This extended PFI was achieved without additional aggressive interventions such as salvage surgery, re-irradiation, second-line bevacizumab, or tumor treating fields (TTF). The patient continued to receive standard concurrent chemoradiotherapy with temozolomide and underwent weekly mEHT sessions as the only supplementary therapy.

During this time, the patient’s KPS remained high, and she demonstrated no significant disease progression on follow-up imaging. MRI scans indicated stable disease and a gradual reduction in tumor size and surrounding edema (as shown in **Figures 3**, **4**). Importantly, the patient’s PFI extended beyond expectations without requiring further invasive or aggressive treatments, highlighting the potential impact of continuous mEHT in maintaining disease control.

This outcome suggests that mEHT may have contributed to delaying disease progression when combined with standard GBM treatment protocols. Although this is a single case, the extended survival without progression underscores the need for further investigation into the role of mEHT in glioblastoma management, significantly as a complementary therapy to enhance standard treatment outcomes.

## Discussion

This case highlights the feasibility and potential of using modulated electro-hyperthermia (mEHT) as an adjunct therapy in the management of newly diagnosed glioblastoma (GBM). While most of the evidence regarding the use of mEHT has been in the recurrent setting, the addition of mEHT in this case for a newly diagnosed patient provides preliminary insight into its safety and potential application earlier in the treatment continuum. However, it is important to emphasize that this case does not establish definitive efficacy but rather highlights the feasibility of integrating mEHT with standard treatments such as concurrent chemoradiotherapy (CCRT) and temozolomide (TMZ) for newly diagnosed GBM patients.

In this case, combining mEHT with CCRT and adjuvant temozolomide correlated with a reduction in tumor size and stabilization of the disease. However, it should be noted that this observation is speculative, and it is difficult to conclusively attribute this outcome solely to the addition of mEHT. GBM is a highly aggressive tumor with a poor prognosis, and reductions in tumor size may be influenced by multiple factors, including the effects of chemoradiotherapy, the patient’s MGMT methylation status, and other biological variables. Therefore, while mEHT may have played a role in this case, further controlled studies are necessary to understand the extent of its contribution to tumor reduction and disease control.

The patient in this case demonstrated IDH-wildtype, which is associated with a worse prognosis. Unfortunately, MGMT methylation status was not available, which leaves an incomplete assessment of the patient’s molecular profile. Patients with MGMT-methylated tumors typically respond better to temozolomide, and the absence of this data limits our understanding of the full therapeutic potential in this case. Therefore, it is difficult to determine whether the observed progression-free interval (PFI) was primarily due to mEHT or the effects of standard therapies, such as CCRT and adjuvant temozolomide.

Additionally, the patient experienced notable improvements in symptoms such as headache, speech fluency, and motor function, which improved her overall quality of life. However, it is important to acknowledge that the improvement in symptoms may not be entirely attributable to mEHT, as they could be related to the effects of radiotherapy, chemotherapy, and other aspects of the overall treatment plan. Therefore, while mEHT may contribute to improving patient outcomes, we cannot definitively claim that it was the primary factor responsible for symptom relief in this case.

In terms of survival, the patient has achieved a stable disease state 14 months post-resection, which is a positive outcome in the context of GBM. However, given the typical aggressive nature of the disease, a 14-month survival period should not be considered exceptional. The available literature on mEHT in the recurrent setting has suggested that it may improve progression-free and overall survival, but these findings are largely based on recurrent cases and retrospective studies. As this case was treated in the newly diagnosed setting, it provides an opportunity to investigate whether the early application of mEHT could yield similar or improved results in prospective studies.

The sagittal MRI scans clearly show a gradual decrease in the tumor dimensions, but this could partly be attributed to the retraction of the surgical cavity following resection, rather than solely due to mEHT treatment. The immediate post-operative MRI revealed a substantial resection cavity, and as the tissue healed, the cavity size may have contributed to the observed reduction in the MRI images. Therefore, while the tumor appeared smaller over time, we cannot definitively attribute this effect to oncothermia. More robust imaging follow-up, including tissue-based analyses, would be needed to clarify whether mEHT directly contributed to the decrease in tumor size.

Hyperthermia offers several benefits for treating brain tumors. First, it enhances the effectiveness of conventional treatments like chemotherapy and radiotherapy by increasing the permeability of the blood-brain barrier, allowing for better drug delivery to the tumor site. Second, hyperthermia induces the expression of heat shock proteins, which can stimulate the immune system to recognize and attack tumor cells more effectively. Third, it directly causes thermal damage to cancer cells, leading to their apoptosis or necrosis, while sparing surrounding healthy tissue due to differential heat sensitivity. Fourth, hyperthermia can disrupt the repair mechanisms of cancer cells, making them more susceptible to radiation- and chemotherapy-induced damage. Finally, when combined with immunotherapy, hyperthermia can enhance the immune response against the tumor, improving overall treatment outcomes (11, 12). Further research and clinical trials are warranted to validate these findings and optimize treatment protocols (11, 13).

Current literature on the use of mEHT in GBM primarily focuses on patients with recurrent disease, where its efficacy has been suggested in retrospective studies as a complementary therapy to standard care (10, 14). However, there is limited evidence for its use in newly diagnosed patients. This case demonstrates the feasibility of integrating mEHT early in the treatment course, alongside chemoradiotherapy and temozolomide, without adding significant toxicity. Nevertheless, without tissue analysis post-treatment or additional biomarkers of response, it is impossible to definitively conclude the effectiveness of mEHT in prolonging survival or improving outcomes. This case underscores the importance of conducting clinical trials to explore mEHT’s role in enhancing therapeutic efficacy in the newly diagnosed setting.

One of the key aspects of mEHT treatment is optimizing power delivery to ensure therapeutic efficacy while maintaining the safety of normal brain tissue. In this study, we maintained power levels below 80 watts, a setting that has been shown in previous clinical trials to be safe and effective for glioblastoma treatment. Clinical evidence suggests that power levels at or below 80 watts selectively target tumor cells while keeping normal brain temperatures below the threshold of 39.2°C, thereby minimizing the risk of thermal damage to healthy tissue (8, 9). Additionally, maintaining this power range optimizes tumor perfusion, enhances oxygenation, and increases radiosensitivity by inhibiting DNA repair processes. This approach is supported by studies demonstrating that lower but sustained hyperthermia temperatures (40–42°C) effectively sensitize tumor cells to radiation and chemotherapy without causing significant neurotoxicity (15).

In conclusion, while this case demonstrates the safety and feasibility of combining mEHT with standard GBM treatments, it does not provide conclusive evidence of its efficacy in prolonging survival or improving disease control. More extensive clinical trials with appropriate controls, biomarkers, and long-term follow-up are necessary to fully understand the potential benefits of mEHT in newly diagnosed GBM patients.

## Data Availability

The original contributions presented in the study are included in the article/supplementary material. Further inquiries can be directed to the corresponding authors.
